# An Affordances-Based Approach and Scoping Review of Virtual Reality Applications in Forensic Behavioral and Mental Health Assessment and Treatment

**DOI:** 10.1007/s10802-025-01383-1

**Published:** 2025-10-24

**Authors:** Esther C. A. Mertens, Jean-Louis van Gelder

**Affiliations:** 1 Department of Criminology, Max Planck Institute for the Study of Crime, Security, and Law, Freiburg im Breisgau, Germany; 2 Netherlands Institute for the Study of Crime and Law Enforcement, Amsterdam, the Netherlands; 3 Institute of Education and Child Studies, Leiden University, Leiden, the Netherlands

**Keywords:** Virtual reality, Affordances, Assessment, Treatment, Forensic, Scoping review

## Abstract

Immersive Virtual Reality (VR) offers plentiful opportunities for behavioral and mental health assessment and treatment, such as overcoming spatial restrictions, increasing treatment motivation, and reducing participant risk. We examine these opportunities through the lens of ‘affordances’, which refers to the specific possibilities a technology offers when users interact with it. This lens provides a frame of reference that can help researchers better understand and exploit the opportunities VR offers for the design of assessments and treatments, boost their effectiveness, and provide a shared vocabulary across disciplines. We illustrate the utility of this approach with a scoping review of VR applications in forensic settings. Most of the 25 studies that were included used multiple VR affordances. While all studies employed transportation (100%), i.e., sensory immersion in a virtual environment that differs from the current physical environment, fewer utilized transformation (30%), i.e., user-embodiment in avatars with characteristics that differ from their own. The proposed affordances-based approach offers a transdiagnostic and user-centered approach to guide development and design of VR-based assessment and treatment across diverse mental health domains. It provides a shared conceptual approach for the organization of intervention components as well as for communication between stakeholders, facilitating the conversion of an idea into a full intervention.

## Introduction

Immersive Virtual Reality (VR) has shown considerable promise in behavioral and mental health assessment and treatment across a range of disorders, including anxiety disorders, phobias, eating disorders, gaming addiction, and disruptive behavior (Blanco et al., 2024). However, due to its experiential nature, identifying the unique possibilities VR offers for assessment and treatment can be challenging. In this article, we adopt the vocabulary of affordances to help identify and exploit the opportunities of this technology. We introduce six key affordances of VR: Transportation, transformation, transfer, tracking, tracing, and tailoring. Together, they capture the core ways in which users can interact meaningfully with VR in therapeutic contexts. We illustrate the relevance of this affordances-based approach through a scoping review of VR applications in forensic settings, although it applies to behavioral and mental health assessment and treatment interventions more broadly. Below, we begin by briefly explaining the concept of affordances before detailing how it can be usefully applied to VR-based assessment and treatment.

### VR Affordances

Gibson (1979) coined the term “affordances” to denote what an environment can *offer*, *provide* or *furnish* to individuals (human and non-human)—i.e., what it enables them to do. Importantly, affordances do not refer to specific properties of an environment, object, or individual, but instead manifest in the *interaction* between the individual and features of their environment (Chemero, 2003). For example, a door only affords opening if an individual interacts with it, that is, if they can reach and operate the handle (Gaver, 1991). The term affordance was later adopted by Norman (1988) in the field of human-computer interaction, where *technology affordances* refer to the perceived possibilities a specific technology provides in the interaction between users and this technology (Norman, 1988). Drawing on the outlined body of work and terminology, we apply an affordances-based approach for identifying how users can interact with VR in the context of behavioral and mental health assessment and treatment. We refer to this approach as *VR affordances*.^1^

Thinking about the implementation of technology in assessment and treatment in terms of affordances, rather than capabilities, characteristics, or opportunities, has multiple advantages. First, an affordances approach shifts the focus from the technology itself to the *interaction* between the technology and the user. This user-centered perspective enables developers to align intervention design with the way users interact with it to achieve the therapeutic objectives, thereby leveraging VR’s specific affordances and maximizing its potential. This alignment can support the creation of new assessments and treatments as well as the optimization of existing ones. For example, it enables a systematic evaluation of whether VR affordances are being fully utilized and helps identify opportunities to better capitalize on specific affordances.

Second, linking intervention goals to affordances rather than specific methods enhances both the *consistency* and *generalizability* of findings regarding effective intervention approaches (Steffen et al., 2019). To illustrate, VR currently enables the affordance of ‘transportation’ by immersing users in a virtual environment through VR goggles. However, this particular method may become obsolete as technology evolves. While the affordance of transportation remains valuable for intervention, emerging technologies—such as augmented reality (AR)—may better support it in the future. By centering intervention development around affordances instead of fixed methods, effective strategies can be more easily adapted across technology types, ensuring that knowledge of intervention approaches remains relevant in a rapidly changing digital landscape.

Third, adopting the term ‘affordances’ provides a *shared vocabulary* that facilitates clearer communication across disciplines, such as intervention research and computer science. Currently, intervention researchers describe VR affordances using various terms, including ‘characteristics’, ‘capabilities’, ‘opportunities’, and ‘possibilities’, while in design and computer science the term ‘affordances’ is consistently used. Standardizing terminology across fields not only improves communication and collaboration but also minimizes the potential for misinterpretation and conceptual ambiguity.

### Present Article

This article is divided into two parts, combining conceptual synthesis with practical illustration. In the first part, we elaborate on six affordances of VR—transportation, transformation, transfer, tracking and tracing, and tailoring—that are particularly relevant to behavioral and mental health assessment and treatment (see Table 1 for definitions). These six affordances have previously appeared isolated and scattered in the literature on VR discussing its features, strengths, and possibilities. We bring them together and frame them as affordances that operate as elements within a larger context. Note that we do not intend the list of six affordances to be exhaustive; additional affordances may emerge and can be added.

**Table 1:** The six VR affordances and their definitions

Affordance	Definition
Transportation	Sensory immersion in a virtual environment that differs from the current physical environment.
Transformation	User-embodiment in avatars (i.e., user-controlled virtual humans) with characteristics that differ from their own.
Transfer	Generalizing skills, behaviors, and cognitions acquired in the virtual environment to the real-world.
Tracking	Passively measuring behavior, attention, and affective states.
Tracing	Observing and recording (near) real-time behavioral processes.
Tailoring	Personalizing the VR experience to the individual needs of users.

The above approach provides the basis for the second part of the article, where we illustrate how the proposed VR affordances can be applied to VR-based behavioral and mental health assessment and treatment. To showcase the approach’s utility, we conducted a scoping review of the literature on VR applications within the forensic setting, both outpatient and residential, and examined the extent to which these leveraged the six affordances. Where relevant, we further distinguished subcategories within each affordance to capture nuanced differences. We focus on forensic settings because these settings amplify the kinds of constraints—such as limited freedom and restricted access to real-world settings—for which VR affordances can be particularly valuable.

We conclude by discussing the broader relevance of the affordances-based approach for VR-based assessment and treatment, and address salient points concerning VR use in forensic assessment and treatment, including current gaps and recommendations for future research. Although we specifically focus on the forensic setting to illustrate this approach, the different affordances are relevant across behavioral and mental health domains.

#### The Forensic Setting

In forensic settings, accurate assessment and effective treatment of behavioral and mental health are pivotal. Accurate assessment is important for identifying risk and protective factors, setting treatment goals, and informing decisions about probation and reintegration. Effective treatment is important for successful rehabilitation and reintegration into society, as well as for the prevention of recidivism (Leach & Powell, 2020; Penn & Thomas, 2005).

Forensic assessment and treatment target a heterogeneous population and take place in a challenging context. Compared to the general population, the forensic population shows more behavioral problems, more difficulties with emotion regulation, and a higher prevalence of mental disorders (Penn & Thomas, 2005). This forensic population is typically less motivated to engage in treatment and often has lower cognitive abilities. As a result, individuals may struggle to follow complex instructions or assignments that require high levels of abstract reasoning or reflection (Kip et al., 2019). Moreover, court-imposed restrictions, such as freedom restrictions, or mandated (types of) confined treatment (Roggeman et al., 2021), can isolate this population from the rest of society, which makes it impossible to observe or practice behavior in the naturalistic settings where such behaviors would normally occur, adding to reintegration challenges (Kip et al., 2019).

We argue that some of these challenges can be mitigated through the affordances of VR. For example, VR can virtually transport users with restricted freedom to a broad variety of relevant real-world scenarios without the need for physical presence in these locations (Blanco et al., 2024).

### Six VR Affordances for Assessment and Treatment

#### Transportation

Arguably, the defining characteristic of VR is its ability to perceptually immerse users in a simulated environment. If properly designed, VR enables users to momentarily disengage from their physical and psychological realities and become absorbed in the virtual world (Cornet & Van Gelder, 2020). This experience of cognitive and emotional engagement is known as *transportation* (Green et al., 2004; Slater & Sanchez-Vives, 2016). While transportation also applies to other types of media, such as books, television, video games, and film, VR is particularly effective in evoking it. Unlike traditional, often linear media, in which the user remains a passive observer of the unfolding narrative, VR is interactive, enabling users to influence the unfolding scenario and course of events (Van Gelder, 2023). Its immersive nature—where the virtual environment completely envelops the user and replaces the physical one—further enhances the sense of transportation. By contrast, non-immersive digital environments, such as computer or smartphone screens, can easily be ignored or be looked away from, breaking the illusion of being ‘there’ (Van Gelder et al., 2019; Gelder et al., 2022). As a result, the experience of presence in VR is more robust, with users often feeling as though they are truly part of the virtual world and accepting it as real. Research shows that psychological transportation can occur even on very short timescales, sometimes within seconds, and leads users to react to the virtual environment as if it were an actual one (e.g., Pan & Hamilton, 2018; Slater & Sanchez-Vives, 2016).

##### Transportation in Forensic Assessment and Treatment

Transportation can be exploited to create environments or situations that would be dangerous, impossible, counterproductive, expensive, or unethical in the real world (Van Gelder, 2023; Wang & Bailenson, 2024). VR enables users to ‘live’ the experience rather than merely imagine it (Roggeman et al., 2021), while avoiding the risks and ethical constraints associated with their real-world equivalents—for example, entering combat situations, performing surgical procedures on a patient, or exposing individuals who have committed sexual offenses against children to actual children to practice coping skills.

In forensic settings, both treatment and assessment may benefit from patients getting exposed to scenarios linked to their problem behavior, such as interpersonal violence or substance use. By transporting individuals to virtual situations that resemble those conducive to their problem behavior, VR enables practice in settings that are both safe and ethical. This creates opportunities for behavioral and cognitive training (e.g., anger management, resisting temptation) that are practically impossible to reproduce in the real world.

#### Transformation

In addition to transporting users into immersive virtual environments, VR can also be employed to have users embody virtual characters or ‘avatars’ with characteristics that differ from their own in a perceptually realistic manner (Gonzalez-Franco & Lanier, 2017). In other words, users can virtually step into the shoes of someone else, such as a person of a different gender, a famous individual, or a fictional character like a superhero, etc. This process, commonly referred to as *virtual embodiment*, involves replacing the user’s physical body with a virtual one (Slater & Sanchez-Vives, 2016).

Virtual embodiment can lead to the temporary illusion of body ownership or ‘transformation’, which can produce nonconscious perceptual and behavioral effects, in spite of users remaining aware that they are in an artificial environment (Bombari et al., 2015; Slater et al., 2010). A related phenomenon, the *Proteus effect*, describes how users’ behaviors and attitudes may be affected by their avatar’s appearance (Yee & Bailenson, 2009). According to this theory, individuals tend to behave in ways that align with the social expectations or stereotypes they associate with their avatar’s visual traits, such as gender, height, or attractiveness.

##### Transformation in Forensic Assessment and Treatment

Transformation can be applied to increase users’ insight into both themselves and others. After undergoing a virtual transformation, users may reflect on their own emotions, motivations, and behavior from that other person’s perspective, potentially gaining novel insights into themselves, such as why they act a certain way or how their behavior is perceived (e.g., Falconer et al., 2016). Transformation can also deepen understanding of others’ emotions, motivations, and behavior (Yee & Bailenson, 2009). By embodying an avatar with characteristics different from their own, users can gain first-hand experience of what it might be like to be that person. In turn, this can cultivate empathy and understanding for that person and their situation.

For forensic patients, transformation can be particularly useful, as it can support both visual and cognitive aspects of abstract reasoning and reflection—two abilities this population struggles with (Kip et al., 2019). For example, in traditional schema-focused treatment, patients move between two chairs and engage in a dialogue between different parts of themselves. This technique enables them to externalize and work through internal conflicts, develop a greater sense of self-awareness, and cultivate a more compassionate and accepting relationship with themselves, resulting in improved emotion regulation, better relationships, and enhanced overall well-being. Using VR for this form of therapy could involve avatars representing the different identity parts, with the patient alternating between embodying these parts. This not only reduces the cognitive burden of having to imagine each perspective, but also enhances engagement by allowing interaction with human-like avatars instead of empty chairs (e.g., Ganschow et al., 2021, 2024).

#### Transfer

VR affords the creation of artificial experiences in real time in which users can be immersed and interact as if in the real world (Botella et al., 2017). For example, users tend to respond to virtual humans, or avatars, in ways that resemble real-life interactions, as the brain processes these virtual agents similarly to real people (Gonzalez-Franco & Lanier, 2017). As a consequence, people adhere to social norms, such as maintaining interpersonal distance, and show complex social behaviors, including shy users responding in timid ways (Gonzalez-Franco & Lanier, 2017).

Because users tend to treat VR experiences as real, behaviors, cognitions, and skills acquired or practiced in a virtual environment can *transfer* to real-world contexts due to the psychological relevance of the virtual experience (Botella et al., 2017). In this sense, the affordance of transfer refers to VR’s potential to support learning and behavioral change that extends into users’ daily lives, rather than limiting its effects to the virtual world.

##### Transfer in Forensic Assessment and Treatment

The tendency of users to behave in virtual environments in similar ways as they do in the real world makes VR a valuable tool for both assessment and treatment. Behavior observed in VR is less susceptible to manipulation by users than self-reported behavior obtained through surveys (Leach & Powell, 2020). By placing users in various (ecologically valid) virtual situations, practitioners can assess the extent to which individuals are able to apply newly acquired skills—for example managing anger or dealing with provocation. VR also enables users to repeatedly practice (new) skills in virtual environments to facilitate their generalization to other contexts. These virtual experiences can be controlled to establish optimal and safe conditions to assess or practice skills (Ticknor, 2018). Virtual objects can be programmed, and avatar behaviors manipulated to suit specific assessment or treatment goals. For example, users can practice to regulate their emotions in settings that resemble their real-life triggers (e.g., a bar setting) with avatars that cannot be harmed. Additionally, avatars do not fatigue, allowing for endless repetition and rehearsal of the same scenario (Wang & Bailenson, 2024).

For practitioners in forensic settings, this affordance has the potential to provide a more accurate indication of a patient’s prospective behavior in society, which can inform critical decisions, such as decisions on release or probation (Roggeman et al., 2021). For patients, it offers a hands-on, experiential learning experience well suited to their needs and characteristics, allowing them to practice skills that are important for a successful reintegration after release. At the same time, the immersive and interactive nature of VR makes the experience engaging (Ticknor, 2018), which may help increase treatment motivation.

#### Tracking and Tracing

VR systems can simultaneously and unobtrusively measure, i.e., track, a variety of behaviors, as well as capture attentional and affective states. These data can be collected without users’ awareness, making the data less vulnerable to biases such as social desirability, demand characteristics, or deliberate manipulation. For example, VR systems can register physiological and behavioral indicators such as pupil dilation, eye gaze, gait, body and spatial movement, as well as speech and vocal expressions (Leach & Powell, 2020; Wang & Bailenson, 2024). These measurements can be combined with other types of technology using sensors that capture physiological states, including heart rate, skin conductance, and/or cortisol level. Together, these multimodal data allow for detailed and simultaneous recording of implicit, natural behaviors (Pan & Hamilton, 2018).

In addition to *tracking*, VR enables the collection of data that can support ‘process tracing’, which provides insights into intermediate stages of decision-making and/or behavior, rather than merely recording the relation between input (e.g., the virtual environment) and output (e.g., decisions, behavior) (Herrmann, 2025). We use the term *tracing* to refer to the affordance of observing behavioral processes in (near) real time (versus end-state registration). By immersing users in a VR experience, it becomes possible to observe behavior as it unfolds, identifying the chain of events or triggers that underly certain behaviors.

##### Tracking and Tracing in Forensic Assessment and Treatment

Data collected through tracking and tracing go beyond what can be captured using surveys and other self-report methods, and can reveal underlying psychological and behavioral processes relevant to assessment and treatment, such as attentional focus or aggression. For example, data on where users direct their gaze in the virtual environment and for how long can identify which elements they pay attention to and also which they disregard, thereby offering insight into perceived relevance or salience of elements. Similarly, users’ spatial movement patterns in the virtual environment can reveal preferences, avoidance tendencies, or habitual behaviors (e.g., Sergiou et al., 2024).

Furthermore, tracking and tracing through VR mitigate practical limitations associated with in vivo observations and treatment. Coding behavior in real-world or video-recorded settings is a highly resource-intensive, time-consuming process that often requires multiple coders (see Van Gelder, 2023). By contrast, VR systems afford the automatic and continuous registration of different types of behavior, such as gaze direction or position in space, without the need for human intervention. Because the observer is not part of the experience, behavior recorded in VR is also less prone to bias, such as social desirability (Leach & Powell, 2020). In addition, VR enables the creation of multiple scenarios, which allows behavior to be observed across diverse contexts (Blanco et al., 2024).

In forensic settings, tracking and tracing can provide practitioners with detailed insights into patients’ reactions to certain environments or triggers (Kip et al., 2019). They can use this knowledge to deliver feedback to correct behavior, resolve misunderstandings, and reinforce adaptive behavior (Ticknor, 2018).

#### Tailoring

Rather than providing a uniform or ‘blanket’ approach in which all users receive the same experience, VR affords *tailoring*, that is, the personalization of the virtual experience to better align with characteristics, needs, and learning goals of individual users. Tailoring can enhance engagement and boost effects by adjusting various features of the VR experience, such as the difficulty level. Thanks to their interactive nature, VR experiences can also be dynamically modified during a VR experience itself, for example by adjusting the content based on users’ reactions and responses or by modifying the behavior of virtual agents in response to user input.

##### Tailoring in Forensic Assessment and Treatment

Users differ widely in characteristics that are related to assessment and treatment effectiveness, including motivation, age, skill set, and cognitive ability. As with conventional methods, tailoring increases the fit between assessment or treatment and user characteristics. For example, VR can simulate scenarios that are challenging for a specific user, adjust task difficulty to match user capabilities, or evoke treatment-relevant behaviors through virtual agents (Ticknor, 2018).

This affordance is pivotal for the forensic setting given the large heterogeneity in this population. Tailoring can support adaptation to diverse individual profiles, including varying risk factors, emotional and cognitive triggers, mental health disorders, and specific offense types (Kip et al., 2019).

### Scoping Review

To illustrate the six affordances described above and how they are applied, we evaluated the current state of the use of VR applications for behavioral and mental health assessment and treatment within forensic settings in a scoping review. Given that this field of research is still in its early stages, we included not only VR applications already implemented in the forensic setting, but also those in developmental phases, as well as applications proposed by their authors as relevant to forensic populations (but that could also be implemented among other populations). With this comprehensive approach, we aimed to provide a thorough and up-to-date overview of existing VR initiatives and to assess which affordances are already capitalized upon in the field and which are possibly underused.

While the review includes VR applications targeting both adults and adolescents, we hold a particular interest in VR applications targeting youth. Young people are often considered digital natives, accustomed to environments in which technology is a fixed, integrated, and important part of life, and typically demonstrate high levels of digital competence (e.g., operational skills, information processing, and interaction; Pongrac et al., 2025). Additionally, adolescence is a critical developmental phase indicated as a window of opportunity for intervention. It is marked by dynamic changes in, and maturation of, neural, biological, and psychosocial functioning which increases sensitivity to environmental factors making adolescents particularly receptive to treatment and rehabilitation efforts (Sisk & Gee, 2022).

## Method

The scoping review is reported following PRISMA-ScR guidelines (Tricco et al., 2018) and was preregistered on the Open Science Framework (OSF Registries | A Scoping Review on the Use of VR in Forensic Assessment and Treatment) on 13 May 2024.

### Eligibility Criteria

We sought to include descriptions of behavioral and mental health assessments and treatments targeting a forensic population. A forensic population was defined as individuals who are, or have been, in contact with the criminal justice system, both outpatient and residential. Studies were eligible for review when (1) they described an assessment or treatment focused on improving behavioral or mental health outcomes (reviews were excluded); (2) the assessment or treatment was aimed at a forensic population; (3) VR was used during the assessment or treatment; and (4) the study was written in the English, Dutch, German, French, or Italian language.

### Literature Search

The following databases were searched on 15 May 2024 (search updated on 27 January 2025) without time period restrictions: PubMed, PsycInfo, and Web of Science. Search terms were used to elicit VR applications (e.g., “virtual reality”, VR) and the forensic setting (e.g., “correctional institutions”, forensic). The complete search string is available in the online preregistration.^2^ The search resulted in 5,191 studies on PubMed, 9,220 studies on Web of Science, and 3,109 studies on PsycInfo. After removing duplicates, 14,521 unique studies remained. Additionally, reference lists of included studies and relevant identified reviews and meta-analyses were searched for additional studies, which resulted in one additional study. Grey literature was included by (1) scanning the contributions included in the special issue ‘Bringing technology to justice-involved youth’ in *Research in Child and Adolescent Psychopathology* (https://link.springer.com/collections/gbheigheah); and (2) searching www.techwijzerFZ.nl^3^ to identify current VR assessments and/or treatments that may not have been published in academic journals. This yielded another two studies.

**Fig. 1:**
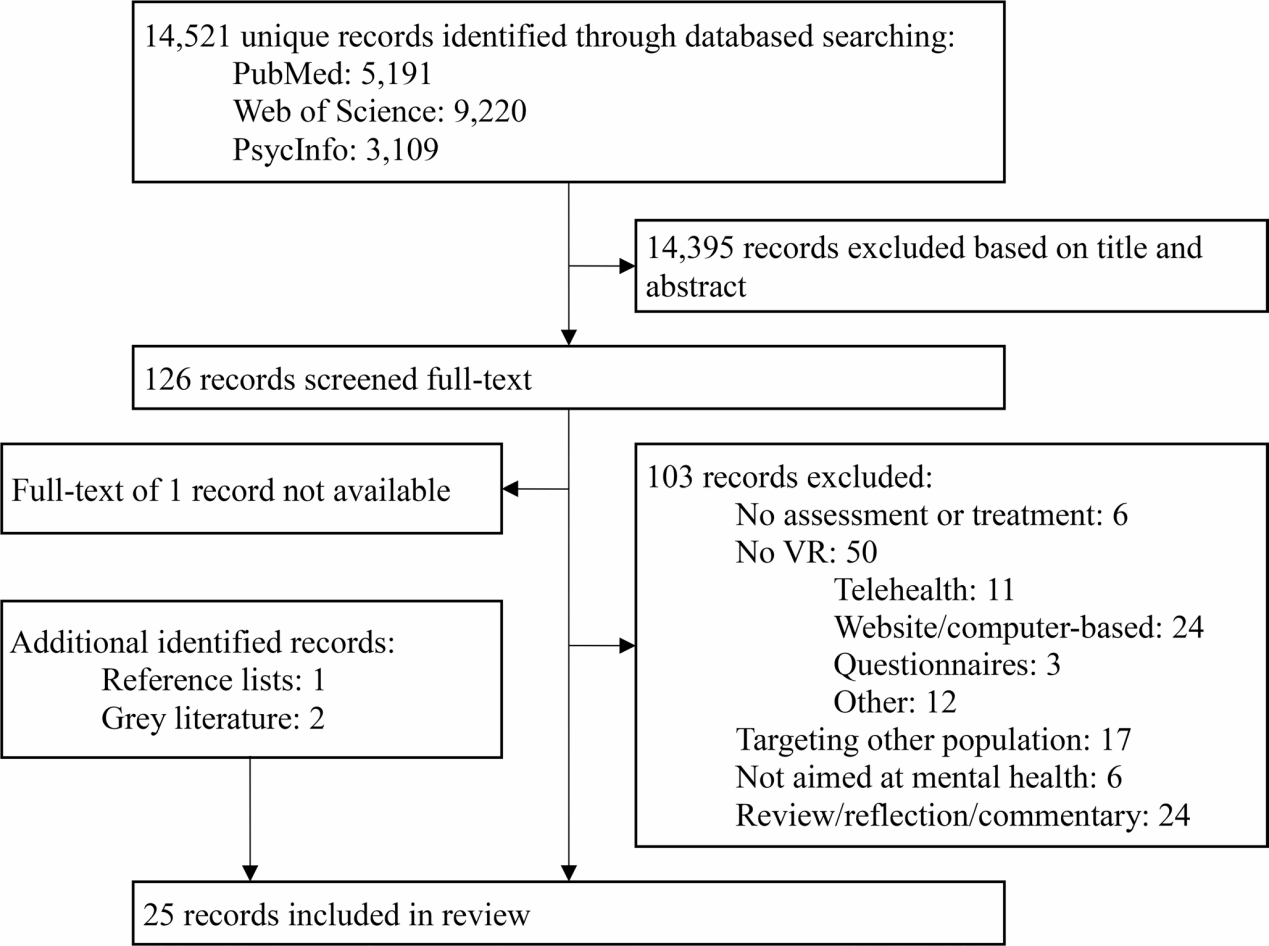
Flow diagram identified studies

Study inclusion proceeded in two phases. In the first phase, studies were screened for eligibility on the basis of title and abstract by the first author based on the inclusion criteria. This resulted in the exclusion of 14,395 studies (99%). In the second phase, the full-text of the remaining studies was assessed for eligibility by the first author, which resulted in an exclusion of an additional 103 studies (82%). See Fig. 1 for the flow diagram.

### Data Extraction

We extracted the following information from the 25 included studies: (1) name of the assessment or treatment; (2) aim of the assessment or treatment; (3) method and content of the assessment or treatment; and (4) population targeted (including age). After coding this information, we extracted the VR affordances that were utilized in the assessment or treatment.

VR affordances and the relevant subcategories were extracted by the first author using deductive/inductive hybrid thematic analysis (Proudfoot, 2023). First, the author familiarized herself with the material by organizing descriptive information about the VR applications into a table, which served as the basis for theme coding. For the deductive thematic analysis, the theoretical affordances framework described above served as a guide to code themes, i.e., affordances. Since none of the articles used the term ‘affordances’, these were inferred from the description of the VR application by the author. For the inductive thematic analysis, the goal with which an affordance was applied in each VR application was labeled. These inductive codes revealed patterns of clustering in these purposes, which were subsequently categorized into broader themes resulting in multiple subcategories per affordance.

## Results

### Descriptives of the Included Studies

Most included studies were published between 2018 and 2025, except for one (Renaud et al., 2014), and conducted or described in the Netherlands (*n* = 12), Sweden (*n* = 3), United States of America (*n* = 3), Germany (*n* = 2), Spain (*n* = 2), Italy (*n* = 1), Canada (*n* = 1), and Saudi Arabia (*n* = 1). Together, the 25 studies described 20 different VR applications of which most involved a treatment (*n* = 16, 80%) and four (20%) an assessment. The psychological and behavioral domains addressed by the assessments and treatments varied. Multiple assessments and treatments focused on aggression (*n* = 6), social and societal skills (*n* = 4), relaxation (*n* = 2), and emotion perception and regulation (*n* = 2). Prosocial behavior (*n* = 1), coping skills (*n* = 1), self-defeating behavior (*n* = 1), cognitive distortions (*n* = 1), sexual arousal profiles (*n* = 1), and paranoia (*n* = 1) were the focus of only one assessment or treatment. Furthermore, most of the included studies targeted adults (> 18 years old; *n* = 18, 90%), while only two (10%) focused on adolescents (< 18 years old). The data extracted, including which VR affordances were leveraged in each VR application, are presented in Table 2.

**Table 2:** Overview of the 25 included studies describing 20 different VR assessments or treatments for the forensic setting

Authors	Sample	Name VR application	Aim	As/Tr	Affordances
Alshaer (2023)	Male inmates from Makkah prison (*N* = 46; age *M* = 23 years)	VR facilitated Practical Educational and Training Services	Rehabilitation to reduce recidivism and promote prosocial behavior in criminal justice settings	Tr	A VR environment designed to reassemble engine components using the VR controller. The system provided visual cues in the form of distinct colors of the engine parts, strategically guiding participants on the appropriate sequence of assembly steps. **Transportation** to a car workshop environment **Transfer** of skills by showing real-looking engine parts and highlighting the part needed next
Barbe et al. (2023)	No sample (description of the treatment)	Open-source VR training framework	Practice conversation skills in a controlled and immersive VR environment	Tr	Virtual characters with different biographies were developed with which conversation using natural language was possible. Avatars’ emotional expressions could be matched to the conversational situation. The framework allowed for interchangeable content that applies within the criminal justice system. **Transportation** to environment with selected type of virtual character **Transfer** of skills by practicing conversation skills through using natural language **Tailoring** of virtual characters to the needs of the participant
Claborn et al. (2024)	Men and women inmates at a correctional facility who were classified as minimum custody (*N* = 22; age *M* = 32.47 years)	Green Meadows scenario from the Nature Treks VR game	VR tools to deliver substance use treatment among people who use drugs and are incarcerated. The scenario is designed to create a relaxing emotional state.	Tr	An immersive 3D experience in a relaxing green meadow. The participant could move through the environment and create different aspects to the experience, such as seeing animals, moving through water, controlling the weather and time of day. Elements within the environments reacted procedurally to the audio and visual cues. **Transportation** to a relaxing environment **Tailoring** of the environment by the participant while in VR
Fromberger et al. (2018)	Non-offenders (*N* = 7; age *M* = 26.00) and men who conducted sexual offences (*N* = 6; age *M* = 47.67 years)	VR-tool for the behavioral monitoring of sexual offenders in risk situations	The possibility for practitioners to monitor the behavior of men who conducted sexual offences and test their decisions on unsupervised privileges without endangering the community, as high-risk virtual situations require the ability of the participants to perform adequate coping strategies.	As	Phase 1, participants rated avatars (male/female, adult/child) on attractiveness based on ‘viewing time’ (passively collected duration time of looking at the avatar) and filling in the attractiveness rating in VR. Phase 2 had five parts: (1) tutorial and training (do groceries), (2) baseline condition (do groceries + unattractive avatar that makes contact and reacts to chosen action of ppt), 3–5) three risk scenarios (do groceries + attractive avatar with increased difficulty to avoid this avatar). **Transportation** to a supermarket to mimic a daily situation **Transfer** of the coping skills learned in therapy to a virtual daily situation **Tracking** through usage data to determine the time participants looked at an avatar and rate its attractiveness to determine what the most attractive avatar is for this participant –objective measure in addition to subjective rating **Tracing** as the therapist can directly observe the behavior of the participant in the virtual environment (and provide feedback through a screen in the virtual environment if wanted) **Tailoring** as the response of the avatar depended on the reaction the participant chose (out of five possibilities)
Renaud et al. (2014)	22 male participants having admitted to engaging in inappropriate sexual conduct with minors (*N* = 22; age *M* = 43.5 years) and non-deviant male participants as control group (*N* = 42; age *M* = 40.7 years)	VR modality to assess sexual arousal profiles and deviance differentials indicative of sexual interests	Computer-generated stimuli presented in virtual immersion to generate sexual arousal profiles representative of sexual interests and deviance differentials characteristic of the presence of previous problematic sexual behavior.	As	Computer-generated stimuli of 3D avatars depicting realistic naked human beings of which the features could be adjusted (both physical body and clothing/naked). Five avatars were presented: (1) adult male, (2) adult female, (3) prepubescent male, (4) prepubescent female, (5) neutral stimulus. Each presented with a neutral emotional attitude for the duration of 90 s. The VR hardware included an infrared system for pupil recognition and gaze direction analyses providing real-time access to the exact gaze location of a participant while exploring the characteristics of a visual stimulus **Transportation** to virtual environment designed to rate the attractiveness of an avatar **Tracking** of pupils and gaze direction to assess where the participant is looking at to track exploration of the avatar as well as for ensuring visual attention to the stimulus (i.e., avatar)
Klein et al. (2020) Klein Tuente et al. (2018) (Protocol paper) Woicik et al. (2023)	Forensic inpatients in VRAPT group (*N* = 64; age *M* = 39.4 years) and in waitlist control (*N* = 64; age *M* = 38.0 years) No sample (description of the treatment) Male detainees (*N* = 17; age *M* = 32 years)	VR Aggression Prevention Training (VRAPT)	Decrease aggression and determinants of aggressive behavior, including anger, impulsivity and hostility	Tr	Each session consisted of a short review of the previous session, clarification of a step of the SIP model, a VR exercise, discussion of the VR exercise, and an evaluation of the progress in learning goals. Sessions 1–5: Exercises on facial emotion recognition and recognizing aggressive behavior of other people. Sessions 6–8: Exercises focused on de-escalating aggressive behavior of others (i.e., avatars) and on regulating physical arousal (i.e., heart rate and skin conductance). Sessions 9–16: All SIP steps were integrated into challenging interactive virtual role-plays. There were three virtual environments where participants could walk around in. Avatars were controlled by the therapist through which they could role-play. The VR experience could be tailored to the specific needs of the participants allowing them to work on their own learning goals and practice with specific triggers. During session 6–15, real-time heart rate and galvanic skin response were measured and displayed at the therapist’s interface for feedback on physical arousal. **Transportation** to different virtual environments resembling (triggering) daily situations **Transfer** by practicing skills in ‘real-life’ situations **Tracking/tracing** by using physiological measures in combination with the VR (multimethod approach: VR and additional technology) **Tailoring** by selecting environments and role-plays based on treatment needs and specific risks for the participant
Gonzalez et al. (2022) Ivarsson et al., (2023)	No sample (description of the treatment) Male violent offenders from medium- and high-security prisons (*N* = 18; age *M* = 29 years)	Revised - VR Aggression Prevention Training (VRAPT)	To provide realistic and safe environments for participants to practice aggression management. Its purpose is to increase awareness of, and improve control over, one’s own aggression and that of others through social interactions in individually tailored virtual environments. The aim of VRAPT is to increase the participant’s understanding and management of his/her dysfunctional aggressive behaviors through identifying triggers and risk scenarios in a conceptualization with assistance of the VRAPT model	Tr	Four modules: 1) Introducing the VRAPT model, treatment conceptualization, and experiencing the virtual environment through walking around in one or multiple virtual environments. 2) Assignments in the virtual world where the participant is tasked with assessing and discerning avatars’ emotions as expressed in facial expressions, and practicing recognition and management of own physiological reactions when confronted with an aggression-provoking role-play in the virtual environment. 3) Skills training: Practices social skills and management of own and others’ aggression through individually tailored therapist-led role-plays. In this module (and part of 2nd module) physiological measures are used to assist therapist and participant in recognizing physiological reactions. 4) Evaluation: Summarizing learning outcomes (not in VR). **Transportation** to different virtual environments resembling (triggering) daily situations **Transfer** by practicing skills in ecologically valid environments **Tracking/tracing** by using physiological measures in combination with the VR (multimethod approach: VR and additional technology) **Tailoring** by selecting environments and role-plays based on treatment needs and specific risks for the participant
Seinfeld et al. (2018)	Offenders convicted for aggression against a woman (*N* = 20) and control men (*N* = 19)	VR domestic violence scene –in a female virtual body	Change socio-perceptual processes such as emotion recognition in male domestic violence offenders – experiment	Tr	Male participants entered a virtual environment, resembling a hallway of a house, where their body was substituted by that of a virtual female and they went through a process of “embodiment”. From this perspective, they saw a virtual male entering the scene and exhibiting abusive speech and gestures along with a progressive invasion of the victim’s (i.e., the participant’s) personal space. The environment was interactive as the virtual abuser gazed towards the face of the participant shouting “Shut up!” if they spoke, or “Look at me!” if they looked away. **Transportation** to a virtual house where the avatars appear to live **Transformation** of a male into a female experiencing domestic violence
Seinfeld et al. (2023)	Men who had perpetrated intimate partner violence (*N* = 31; age *M* = 41.84 years) and men control group (*N* = 19; age *M* = 40.58 years)	VR domestic violence scene –in a child virtual body	Change emotion recognition, physiological reactions, and attitudes towards violence – experiment	Tr	Participants were immersed in a virtual room with a long hallway where they embodied a virtual child with an age appearing to be 4 or 5 years old. From this perspective, they saw a female avatar entering that showed affection and together they conducted an exercise for rapport building. Next, a male avatar entered the room and began to verbally abuse the female avatar, looked at the eyes of the female avatar, threw a telephone to the floor. If participants interrupted the monologue of the male avatar, the experimenter could trigger the male avatar to say “Shut up!” to the virtual child (i.e., participant). The male avatar walked closer three times, invading the personal space of the female and child avatar. **Transportation** to a virtual house where the avatars appear to live **Transformation** of a male into a child witnessing domestic violence
Carnevale et al. (2024)	Men in treatment (voluntary and judicial) for intimate partner violence (*N* = 46; age *M* = 41.78)	ViDaCS	Help men recognize their violent behavior, to motivate themselves to adhere to perpetrators treatment and to adopt new, not-violent, behavior	Tr	First, participants embodied a male avatar that entered his house and became agitated because he could not find his keys. Then, a verbal conflict with his partner started. After this conflict, time was virtually reversed. Participants now embodied a child avatar (age of the child could be manipulated) that experienced the previous conflict situation between the parents. The participant could choose from multiple behaviors dependent upon the child-avatar’s age (e.g., hide under the desk, call a friend, run away). While participants were in VR, their behavior was observed by a therapist. **Transportation** to a virtual house where the avatars appear to live **Transformation** into a child who witnessed intimate partner violence **Tracing** by observing the behavior of the participant **Tailoring** based on the chosen behavioral responses
Hedström et al. (2023)	Forensic psychiatric inpatients from a high-security forensic psychiatric clinic (*N* = 10; age *M* = 35.8 years)	VR-assisted assessment for paranoid ideation	To assess the level of paranoid ideation	As	Participants walked around in two virtual environments simulating a supermarket and a bus scenario. In the supermarket participants were instructed to do groceries while avatars with neutral faces walked around. Avatars shortly turned their attention to participants if they came close, but no other interaction with avatars was possible. In the bus scenario, participants sat with other avatars in a bus, across a male and female avatar. The female avatar was on the phone and participants could overhear the conversation. At the end the female avatar asked the participants for directions. After participants answered the scenario ends. **Transportation** to neutral environments and relatable to most people **Tracing** by observing the participant’s behavior (physical and verbal expressions) by the therapist who rates the behavior using a structured observation protocol with open questions
Hendriks et al. (2023)	Forensic mental health patients with severe psychiatric problems (*N* = 10; age *M* = 40.4)	VReedom	A VR-assisted authorized leave training – aids patients to (re-)learn skills needed in realistic physical environments, without the necessity of being exposed to real-world situations	Tr	The therapy is based on elements of exposure therapy and mimics real authorized leave with activities like walking outside, going to the supermarket, and interacting with a stranger (an embodied conversational agent). Participants were challenged with behavioral problems and potentially stressful situations which required participants to use coping mechanisms. The goal was to gradually decrease stress levels, and subsequent unwanted behavior, by practicing challenging situations with personalized triggers, tailored to the participant’s specific needs. Session 1: Walk around the clinic and nearby supermarket. Session 2: Triggers introduced in supermarket. Session 3: Difficulty level increases. Session 4: Medium-level confrontations through role-play). Session 5: Repeated role-play at a more difficult level. **Transportation** of the participant to surrounding of the clinic and nearby supermarket **Transfer** of skills by practicing in multiple VR sessions **Tailoring** of the environment and interactions to individual triggers and needs
Klein Haneveld et al. (2023)	6 focus groups with healthcare providers and 13 interviews with forensic patients	DEEP VR	Teaches diaphragmatic breathing which has shown its potential in reducing stress in other populations	Tr	By applying diaphragmatic breathing, the participant “swam” through a fictional virtual underwater environment and explored colorful caves while following a, by artists designed, route. The biofeedback in DEEP provided participants with real-time information about their breathing by using a waistband that measured the movement of their diaphragm and by showing them visualizations of how well they were inhaling and exhaling (e.g., corals that light up in DEEP and visual breathing circles). **Transportation** to a relaxing and visually attractive virtual underwater world **Transfer** of skills by practicing skills in the virtual underwater world **Tracking** of breathing using a waistband – biofeedback (multimethod approach: VR and additional technology)
McGivney et al. (2024)	27 participants who were involved with criminal justice system (*N* = 27; age *M* = 43 years)	Project OVERCOME	The impact of a job interview VR simulation on the emotions, confidence, and self-efficacy beliefs of jobseekers who have been impacted by the criminal justice system	Tr	The VR experience consisted of two main parts: (1) Journeys, in which participants heard the stories of and advice from people who have successfully reentered the workforce post-incarceration, and (2) an interview simulation in which participants received advice from a career counselor and then participated in an interview with a hiring manager. In the interview, participants played the role of Nadia who is applying for a job in an industrial laundry facility after being released from incarceration. The interview simulation used a branched narrative structure in which the participant selected an answer from three to four answer-choices to each interview question. The hiring manager responded based on what the participant answered. At the end of the simulation participants were shown their answers to all the questions and received some feedback on how they should respond in an interview. **Transportation** to a job coach and job interview setting **Transformation** into an example participant for practicing in a ‘case study’ (not as treatment technique) **Transfer** of skills by practicing skills in the virtual world **Tracking** of responses in order to provide feedback at the end of the session **Tailoring** based on the responses of the participants which determined the flow of the conversation
Smeijers et al. (2021) Smeijers and Koole (2019) (Protocol paper)	Forensic psychiatric outpatients with aggression regulation problems (*N* = 51; age *M* = 36.13 years)	Virtual Reality Game for Aggressive Impulse Management (VR-GAIME)	Decrease the level of aggressive behavior of forensic psychiatric outpatients	Tr	Participants were assigned the role of a courier who has to collect packages in a shopping street. In the shopping street, participants were met by avatars who were acting in either an agreeable or disagreeable manner –manipulated for training purposes. Participants were trained to respond with approach behavior to friendly situations and with avoidance behavior to anger-relevant situations. The game had five levels with increasing difficulty. **Transportation** to a shopping street where the game is played **Transformation** into a courier (not as treatment technique) **Transfer** of skills by practicing in the virtual environment **Tracking** of ‘avoidance/approach’ behaviors in response to avatars
Teng and Gordon (2021)	Workshops with incarcerated women (*N* = 6), staff (*N* = 9), formerly incarcerated people (*N* = 9), women in addiction recovery (*N* = 2), and some reentry service providers	VR reentry program	To help incarcerated women practice responding to high-stress reentry situations prior to their release	Tr	There were three 360^0^ scenarios of stressful reentry situations: 1) While waiting outside for your first parole meeting, your best friend got high in her car and offers to buy you a drink to celebrate your release. 2) You are running late for an NA meeting during rush hour at a train station and need to ask several strangers for help buying a ticket 3) You are interviewed for a job at an uncomfortable high-end restaurant and are illegally asked by the manager about your criminal record. Each module had an open ending, on which participants could reflect on the challenge, outcome, and immediate next challenge. **Transportation** from prison to potential stressful situations in society
Van Gelder et al. (2022)	Convicted male offenders (*N* = 24; age *M* = 23.7 years)	FutureU	Increase participants’ ability to imagine themselves in the future and reduce their engagement in self-defeating behavior	Tr	Participants alternated between embodying a present self-avatar and a 10-year-older, future, self-avatar. First, participants embodied their present self-avatar and were presented with several statements related to positive and negative behaviors. They indicated whether these statements applied to them or not. Then, they embodied the future self-avatar to reflect on whether the selected behaviors were beneficial or harmful for their future self by sorting these into different stacks. After returning to embodying the present self, participants received a ‘future-self score’ based on the number of positive and negative behaviors in each stack. The exercise ended with the participant embodying the future self and providing free-format advice, which was played back to the present self. **Transportation** to a virtual ‘time portal’ enabling time traveling **Transformation** when embodying the future self-avatar **Tailoring** of the avatars’ appearance and by letting participants themselves sort the behaviors and reflect on them
Van Wolffelaar et al. (2024) (Protocol paper)	Male patients 18 years or older exhibiting aggressive behavior and a moderate to high risk of recidivism	Responsive Aggression Regulation Therapy in VR (Re-ART VR)	Enhancing the degree of aggression regulation among aggressive forensic outpatients	Tr	Examined the Re-ART modules Controlling Skills, Influence of Thinking, and Handling Conflicts, which are offered in that order and usually last about 3–6 months. The Controlling Skills module focuses on learning control methods, to help individuals take a time-out more easily or stay calm when a time-out is not feasible, and to apply these methods in practice. The Influence of Thinking module focuses on reducing distorting cognitions and applying helping thoughts during difficult situations. The Handling Conflicts module aims to teach various skills necessary to handle conflict constructively, such as communicating appropriately, dealing with authorities and dealing with criticism. Exercises and role plays were provided utilizing VR. For example, anger-provoking situations were simulated, allowing participants to practice skills aimed at preventing the escalation of aggression. The therapist observed the virtual setting and could control avatars’ behavior, emotional expressions, and voice. Additionally, the therapist could pause the experience to directly communicate with the participant in the virtual environment. **Transportation** to different types of virtual environments of daily life **Transfer** of skills learned during therapy to ‘real-life’ situations and practicing these skills in virtual environments **Tailoring** of responses to participants by manipulating avatars
Klein Schaarsberg et al. (2025) Klein Schaarsberg et al. (2022) (Protocol paper)	Adolescents with severe behavioral problems who dropped-out of school and follow a resocialization trajectory at an ambulatory treatment setting (*N* = 5; age *M* = 16.3 years)	Street Temptation	Increasing treatment motivation and cognitive distortions	Tr	Street temptation consisted of two modules. In the first module (3 sessions) participants watched a 360^0^ VR video presenting a fictional situation showing (violent) disruptive behavior in which three characters were involved. Each session revolved around perspective-taking of one of the characters. In the second module (2 sessions), participants chose a personal experience for the perspective-taking assignment. This personal experience was visualized using a VR street view application to transport participants to the location of the concerned situation. In both modules, the therapist can observe the virtual scenario in real-time on an additional screen. **Transportation** to a fictional situation as well as to the location where a personal relevant situation occurred to stimulate mentalization **Tracing** by streaming the VR scenario to an additional screen for real-time observation by the therapist **Tailoring** of the content to personally relevant situations in Module 2
Westerveld et al. (2025)	Boys from a short-term stay group in juvenile detention centers (*N* = 87; age *M* = 17.8)	What’s up?	Measure the origin of reactive aggression, with a focus on hostile intent attribution and low self-control	As	The interactive task consisted of five standardized scenarios that took place at a schoolyard after school time –one neutral scenario and four scenarios that contained social triggers that could elicit aggression. In the scenarios, participants were playing a virtual game in the presence of multiple avatars. These avatars were controlled by the facilitator to provoke participants and respond to their behaviors. After each scenario, participants answered questions about their emotions, thoughts, and motives. During the VR task, participants’ behavioral responses were observed. **Transportation** to a virtual schoolyard **Tracking** of behavioral responses during the VR tasks by the facilitator **Tailoring** the provocations and responses of the avatars to participants’ behaviors

Note. *As *Assessment, *Tr *Treatment

This field of research is still in its infancy as illustrated by studies merely describing the application (*n* = 3), examining feasibility and/or acceptability (*n* = 6), or small-scale pilot studies to gather preliminary evidence of effectiveness (*n* = 10). Only one study (Klein Tuente et al., 2020) included a sufficiently powered sample and used a control condition to assess effectiveness. Nevertheless, across studies, both patients and practitioners generally expressed positive attitudes toward the use of VR (e.g., interest, willingness to engage) and some small positive effects were reported. However, the available evidence remains too limited to draw firm conclusions about the effectiveness of VR assessment and treatment in forensic settings.

### Utilization of the Six VR Affordances in VR-Based Assessments and Treatments

#### Transportation

Given that transportation is a defining characteristic of VR, all 20 (100%) VR assessments (*n* = 4) and treatments (*n* = 16) described in the included studies utilized this affordance. Transportation can serve distinct purposes, however. We distinguish three different categories: (1) environments conceptually matching the VR assignment, (2) environments aimed at practicing specific skills under ecologically valid circumstances, and (3) (photo-realistic) environments simulating existing real-life locations. These categories are not mutually exclusive, although one of these purposes is typically emphasized.

In the first category, environments are not specifically designed to mimic real-life situations, but to provide a relevant context for the assignment with the goal of increasing users’ understanding of its purpose (Mertens & Van Gelder, 2025). For example, if the assignment is assembling a motor block, the environment in which this takes place can be a car workshop (Alshaer, 2023). If the assignment is a breathing exercise, the environment could be designed to generate a calming effect, such as a green meadow (Claborn et al., 2024) or an underwater world (Klein Haneveld et al., 2023). More specific to the forensic setting, the Virtual Reality Game for Aggressive Impulse Management (VR-GAIME; Smeijers et al., 2021; Smeijers & Koole, 2019), which aims to decrease aggressive behavior, places forensic psychiatric outpatients in the role of a parcel deliverer who needs to collect parcels from agreeable and disagreeable avatars and to respond appropriately by respectively approaching or avoiding them. Hence, to conceptually match the assignment, the virtual environment here is a street with shops.

In the second category, users are transported to environments that resemble the real-life environments in which the trained or acquired skills need to eventually be performed. This type of transportation goes beyond merely matching the environment to the assignment, and focuses on maximizing ecological validity. For instance, to help men in treatment for domestic violence recognize their violent behavior and adopt non-violent behavior, ViDaCS (Carnevale et al., 2024) transports them to a living room with a male avatar (embodied by the user), his partner, and a child. An aggression-triggering situation takes place, which starts a conflict between him and his partner. Subsequently, time is reversed, and users now embody the child avatar, witnessing the episode of domestic violence from the child’s perspective. This virtual environment simulates a typical domestic violence situation, creating real-life circumstances for users to practice their skills.

In the third category, users are transported to environments that actually exist in the real world. These environments are often made to look as photo-realistically as possible in order to mimic the real-world location as closely as possible. The emphasis is on creating a user experience that feels real and authentic to bolster users’ role-play and mentalization (i.e., the ability to understand one’s own behavior and that of others in terms of underlying, intentional mental states, such as desires, needs, feelings, attitudes, and intentions; Klein Schaarsberg et al., 2025). In Street Wise (Klein Schaarsberg et al., 2022, 2025), for instance, adolescents with disruptive behavior problems are virtually transported via Google Street View VR to locations where they previously had influential personal experiences, such as the neighborhood where they committed their offense. While recounting the event to a therapist, this transportation supports their mentalization ability. The VReedom intervention (Hendriks et al., 2023) is another example and immerses patients in closed forensic mental healthcare clinics in photo-realistic environments mimicking actual locations around the clinic, such as nearby streets or the local supermarket. Patients can walk around and engage with other avatars (controlled by the therapist) through role-play. This exercise prepares them for authorized leave.

#### Transformation

Six (30%) of the 20 included VR applications incorporated transformation, all for treatment rather than assessment purposes. These applications can be classified into two categories: (1) transformation into a character to conceptually match the assignment; and (2) transformation into a character with a different perspective to facilitate perspective-taking.

In the first category, users embody a character that aligns conceptually with the task or narrative presented in VR. The transformation serves to establish coherence between the assignment and the user’s character. For example, in Project OVERCOME (McGivney et al., 2024), justice-involved adults embody a virtual avatar named ‘Nadia’, who is attending a job interview after being released from incarceration, and participate as Nadia in the job interview.

In the second category, transformation is used as a treatment technique to promote perspective-taking. By stepping into the virtual shoes of a person whose perspective differs from their own, users are exposed to situations designed to deepen their understanding of others’ attitudes, feelings, and behaviors. For instance, Seinfeld et al. (2018) have male domestic violence perpetrators embody a female avatar who is approached by a man progressively displaying abusive speech and physically intrusive behavior in a domestic scene. This experience enables users to experience domestic violence from a first-person perspective of the victim. In a follow-up study, Seinfeld et al. (2023) have domestic violence perpetrators embody child-avatars and witness a scene of domestic violence from this third-person perspective. Initially, the child interacts positively with a female avatar, after which a male avatar enters the domestic scene and starts to verbally abuse the woman and behaves aggressively. In both studies, transformation is applied as a perspective-taking treatment technique which positively affected users’ emotion recognition and attitudes towards domestic violence.

### Transfer

The affordance of *transfer* was identified in 10 (50%) of the 20 included VR applications, of which nine involved treatment and one assessment. In these applications, VR was employed to gain insight into the extent to which (newly acquired) skills would carry over to the real world. For treatment purposes, this typically involved practicing skills in different types of virtual situations intended to support transfer beyond the VR context. Two closely related categories could be identified: (1) practicing (new) skills in different types of situations simulating real-world circumstances; and (2) practicing skills in fictional virtual environments designed to optimally support the utilization of the concerned skill.

In the first category, users practice skills in (different types of) environments that aim to mimic ecologically valid circumstances to support the generalization of these skills to real-world settings. In Virtual Reality Aggression Prevention Therapy (VRAPT; Klein Tuente et al., 2018; Klein Tuente et al., 2020; Woicik et al., 2023), for instance, forensic psychiatric inpatients are targeted with the aim of decreasing their aggression. In a face-to-face setting, a therapist presents the Social Information Processing model, which explains how one’s interpretation and responses to social situations can result in aggressive behavior. In VR, the patients can practice with the cognitive-emotional steps of this model, which are intended to help them cope with provocation by others in non-violent ways and prevent their own aggressive behavior. To generalize these skills to other contexts, there are three different everyday-life environments, i.e., a supermarket, a shopping street, and a café, where they can apply their new skills and interact with other avatars.

In the second category, skills are also practiced in VR to facilitate transfer to the real-world, but here the environments are fictional rather than simulations of the real world. These environments are deliberately designed to optimize the practice of specific skills without mirroring any particular real-world context. For example, DEEP VR (Klein Haneveld et al., 2023) aims to teach diaphragmatic breathing, as this type of deep breathing is related to stress reduction. To this end, users are immersed in a fictional underwater world that becomes more visually rewarding (e.g., corals that light up, colorful fish appear) as a function of diaphragmatic breathing.

#### Tracking and Tracing

*Tracking* and/or *tracing* were utilized in 11 (55%) of the 20 included VR applications, comprising four assessments and seven treatments. Because these two affordances are often intertwined in VR applications, they are discussed together here. We identified three categories: Tracking and tracing via (1) sensors integrated in the VR hardware; (2) observation of users during VR sessions; and (3) additional sensors and wearables connected to the VR hardware.

In the first category, integrated sensors can measure physiological changes, movement, and sounds, such as pupil dilation, gaze direction, head movement, and voices. To illustrate, the VR application of Barbe et al. (2023) provides an environment to practice communication skills in a one-on-one setting. Users engage in conversation with an avatar whose body posture and emotional expression dynamically adjust based on the content of the conversation to mimic realistic communication behavior. Audio input (i.e., user’s voice) is captured via the headset’s microphone, and the software analyzes the emotional content to guide the avatar’s reactions. After the conversation, users receive standardized reports on their communication skills.

In the second category, therapists or researchers observe users while they are engaged in VR. The observer is physically present in the same space, but remains outside the virtual environment. In a VR-assisted assessment of paranoid ideation (Hedström et al., 2023), for instance, therapists observe the behavior of patients referred by a psychiatrist for paranoia assessment while exposed to two virtual scenarios. In the first, they are instructed to purchase milk in a supermarket in the presence of other (neutral) avatars. After finding the milk, users queue at the cashier until the scenario ends after five minutes. Avatars move randomly and do not respond to users; only ambient sounds are present. In the second scenario, patients are on a bus with other (neutral) avatars. A woman seated opposite from the patient engages in a heated phone conversation, and after ending the call, calmly asks the user for directions. Throughout both scenarios, a therapist observes and rates users’ physical and verbal expressions using a structured observation protocol with open questions to assess their level of paranoia.

In the third category, behavior is tracked and traced via sensors and wearables that are connected to the VR hardware and provide additional data streams. These sensors and wearables can measure physiological processes, such as skin conductance, heart rate, and brain activity. These measurements can optionally be used as input for the VR experience, serving as real-time biofeedback. For example, the VRAPT (Klein Tuente et al., 2018, 2020; Woicik et al., 2023), which was described earlier in this article, combines VR with heart rate and galvanic skin response as objective indicators of physical arousal. These measurements are displayed in an interface for the therapist enabling real-time feedback.

#### Tailoring

Twelve (60%) of the VR applications made use of the affordance of *tailoring*, of which one application regarded assessment, and the others treatment. Tailoring could be categorized in two ways: (1) tailoring *before* users enter VR; and (2) tailoring *while* users are in VR. These two categories are not mutually exclusive and are often combined.

In the first category, the virtual experience is tailored to the skills, wants, and needs of users before they enter the virtual environment. This can entail tailoring the nature of the virtual experience (e.g., selecting scenarios that align with situations that the user finds challenging), as well as tailoring the difficulty level of the VR experience to match users’ coping skills (e.g., interactions with neutral avatars versus aggressive avatars). In the VR application of Barbe et al. (2023), users, such as forensic patients, can practice conversational skills by talking to an avatar. This VR application is able to process free speech and natural language, which results in realistic conversations. Prior to entering VR, users select the scenario, avatar, and the avatar’s emotional expressions. Following this tailoring, users can create virtual situations that best match the type of conversational situation they want to practice.

In the second category, the virtual experience is tailored to users while they are in the virtual environment based on their responses and actions. This often involves controlling avatars’ behavior, responses, or emotional expression in reaction to the user’s choices and behaviors. For example, Responsive Aggression Regulation Therapy in VR (Re-ART VR; Van Wolffelaar et al., 2024) aims to enhance aggression regulation of aggressive forensic outpatients by placing them in virtual situations that provoke anger. Therapists control the behavior, emotional expressions, and voices of the avatars to tailor the virtual situations to individual users. Similarly, in What’s up? (Westerveld et al., 2025) the reactive aggression of boys in juvenile detention centers is assessed. The boys play a game in VR with multiple avatars present that provoke them; the facilitator controls the voices of the avatars to tailor the level of provocation.

An example of a VR application that combines both types of tailoring is FutureU (Van Gelder et al., 2022). In one study under this research program, which aims to reduce self-defeating behavior, convicted male offenders are exposed to an avatar representing their 10-year older self, their ‘future self’. Prior to entering VR, two personalized avatars, representing the present and the future self, are created based on a photo of the user’s face, personalized body proportions, and clothing colors matching the user’s outfit (with the user selecting the future self’s outfit). During the VR experience, offenders alternate between embodying their present-self avatar and their future-self avatar. While embodying their present self, participants are asked to sort a range of behaviors into behaviors that apply to them and behaviors that do not. When embodying their future self, they reflect on these behaviors. As a result, the entire experience is tailored to the individual in terms of content, visuals, and procedures.

## Discussion

### An Affordances-Based Approach for Behavioral and Mental Health VR Assessment and Treatment

The current article introduced an affordances-based approach to systematically explore how VR can support behavioral and mental health assessment and treatment. Rather than focusing on static features or technical specifications, this approach centers on what VR *affords* users, emphasizing functional possibilities from a transdiagnostic, user-centered perspective. By grouping and defining the affordances, we offer a structured overview to better understand how VR’s unique capabilities can interact and reinforce each other’s intervention potential.

Intervention developers can use this approach to map VR affordances onto specific intervention components. For each component, it can be described which affordances are utilized and how, in order to operationalize that component and achieve the intervention goal. This procedure can support the optimization of existing assessments and treatments, as well as guide the development of new ones. Additionally, our approach provides a shared lexicon for intervention developers, computer scientists, and programmers, facilitating communication between these stakeholders during the development process.

Moreover, the VR affordances show transdiagnostic relevance. The assessments and treatments included in our scoping review addressed a broad range of behavioral and mental health outcomes, such as aggression (e.g., Klein Tuente et al., 2020), social and societal skills (e.g., Hendriks et al., 2023), and cognitive distortions (Klein Schaarsberg et al., 2025), with content ranging from a fictional underwater world (Klein Haneveld et al., 2023) to grocery shopping in a supermarket (e.g., Hedström et al., 2023). Despite this heterogeneity in both outcomes and content, the affordances-based approach could be applied consistently across the reviewed applications without requiring modification. This transdiagnostic ability underscores the approach’s relevance and applicability for behavioral and mental health care in general.

### Behavioral and Mental Health VR Assessment and Treatment in the Forensic Setting

The use of immersive VR for behavioral and mental health assessment and treatment in the forensic setting is still in its infancy, which makes it difficult to draw firm conclusions about the effectiveness of VR and the underlying affordances. We were only able to include 25 studies reporting on 20 distinct VR applications, the majority of which were in the early stages of development and primarily aimed at treatment (*n* = 16). The identified assessments and treatments addressed a broad range of domains, with a particular focus on aggressive behavior. Notably, almost all assessments and treatments targeted the adult population, despite the potential of VR for adolescents, who are seen as digital natives.

The VR affordances approach applied in this review yielded several valuable insights into how opportunities for assessment and treatment offered by VR are capitalized on in forensic settings. Typically, VR applications combined multiple VR affordances. Transportation was leveraged by all applications, highlighting VR’s potential for use in settings with freedom restrictions, such as prisons and residential mental health institutions. Roughly half of the VR assessments and treatments afforded transfer, tracking and tracing, and tailoring. In some cases, tracking and tracing was achieved through a multimodal approach that combined VR soft- and hardware with additional technologies, such as wearables (e.g., Klein Tuente et al., 2020).

The affordance of transformation emerged in only a few treatments and not in any of the assessments. Although it may be a deliberate decision not to use transformation, the VR treatments that do employ it show that it has potential to change users’ attitudes and behavior, as it stimulates perspective-taking (e.g., Seinfeld et al., 2018; Van Gelder et al., 2022). Unravelling how transformation can be meaningfully applied in both assessment and treatment could be a potentially valuable area for future exploration.

#### Recommendations for Future VR Research in the Forensic Setting

Based on the results of the review, we make two recommendations for future research. First, more research into the effectiveness of VR applications for assessment and treatment in the forensic setting is needed. Most existing studies are still at the early stage of development. Given that the generally positive results regarding feasibility, acceptability, and preliminary effectiveness underscore the potential of VR, further research focused on VR’s added value relative to care as usual and identifying best practices for implementation is pivotal. It would be particularly insightful if this research is guided by the affordances-based approach—for example via a component-based design—to help clarify which affordances are most effective, for whom, and under what circumstances. This can support a targeted implementation of VR, leveraging the full potential of the different affordances.

Second, we recommend an increased focus on VR applications for assessment and treatment that specifically target youth in the forensic setting. As digital natives, young people are technically adept at engaging with technology (Pongrac et al., 2025), and adolescence is a developmental period characterized by increased receptiveness to intervention (Sisk & Gee, 2022), increasing the chance of successful rehabilitation and reintegration with the right assessments and treatments. Given that current efforts have shown limited effects (Pappas & Dent, 2023), VR applications may provide the needed innovation in this domain.

### Limitations

As VR is a fast-developing domain, it is possible that some relevant recent applications for assessment and treatment were not included in this review due to study protocols and/or empirical findings not yet being published at the time of writing this article. We attempted to mitigate this limitation by conducting an extensive systematic search and including grey literature from an online platform aimed at providing an up-to-date overview of initiatives that use technology in forensic settings in the Netherlands. While we may still have missed relevant applications, the scoping review nevertheless provides an extensive overview of the different ways in which VR can be implemented for assessment and treatment purposes in the forensic setting and how affordances are being leveraged.

It is also important to consider ethical and methodological implications when employing VR for assessment and treatment purposes, especially when targeting a vulnerable population such as that in the forensic setting. For a detailed discussion of these considerations, we refer to the contribution of Kip et al. (2025) in this special issue.

### Concluding Remarks

We believe that the affordances-based approach to VR outlined in this article can enhance understanding of the technology and its potential applications in assessment and treatment. By adopting this approach, researchers can advance the field by conceptualizing VR in a comprehensive manner, shifting from a focus on individual features to the broader possibilities that the technology affords. Given the early stage of development in this field, embracing a unified approach can accelerate scientific progress and foster collaboration, support the optimization of intervention strategies, and facilitate communication across stakeholders in research, development, and implementation.

## Data Availability

Materials are available via the Open Science Framework https://osf.io/c9j34/.

## Footnotes

1 We use the term ‘users’ broadly, referring both to people entering the virtual world and people employing the technology, such as practitioners or researchers.

2 The term “technology” was excluded from the search string when searching the database Web of Science, as this term resulted in more than 7,000 additional studies on power industry and secure facilities which were not relevant for the current scoping review.

3 This website provides an overview of Dutch technological applications that are available or interesting for forensic behavioral and mental healthcare and directly applicable to treating and supervising patients.
